# Cellular and Molecular Mechanisms Regulating Retinal Synapse Development

**DOI:** 10.1146/annurev-vision-102122-105721

**Published:** 2024-09

**Authors:** Whitney A. Stevens-Sostre, Mrinalini Hoon

**Affiliations:** 1 Department of Ophthalmology and Visual Sciences, University of Wisconsin–Madison, Madison, Wisconsin, USA;; 2 McPherson Eye Research Institute, University of Wisconsin–Madison, Madison, Wisconsin, USA; 3 Department of Neuroscience, University of Wisconsin–Madison, Madison, Wisconsin, USA

**Keywords:** retina, synapses, mechanisms, organizers, activity

## Abstract

Synapse formation within the retinal circuit ensures that distinct neuronal types can communicate efficiently to process visual signals. Synapses thus form the core of the visual computations performed by the retinal circuit. Retinal synapses are diverse but can be broadly categorized into multipartner ribbon synapses and 1:1 conventional synapses. In this article, we review our current understanding of the cellular and molecular mechanisms that regulate the functional establishment of mammalian retinal synapses, including the role of adhesion proteins, synaptic proteins, extracellular matrix and cytoskeletal-associated proteins, and activity-dependent cues. We outline future directions and areas of research that will expand our knowledge of these mechanisms. Understanding the regulators moderating synapse formation and function not only reveals the integrated developmental processes that establish retinal circuits, but also divulges the identity of mechanisms that could be engaged during disease and degeneration.

## 1. INTRODUCTION

**Figure 1:**
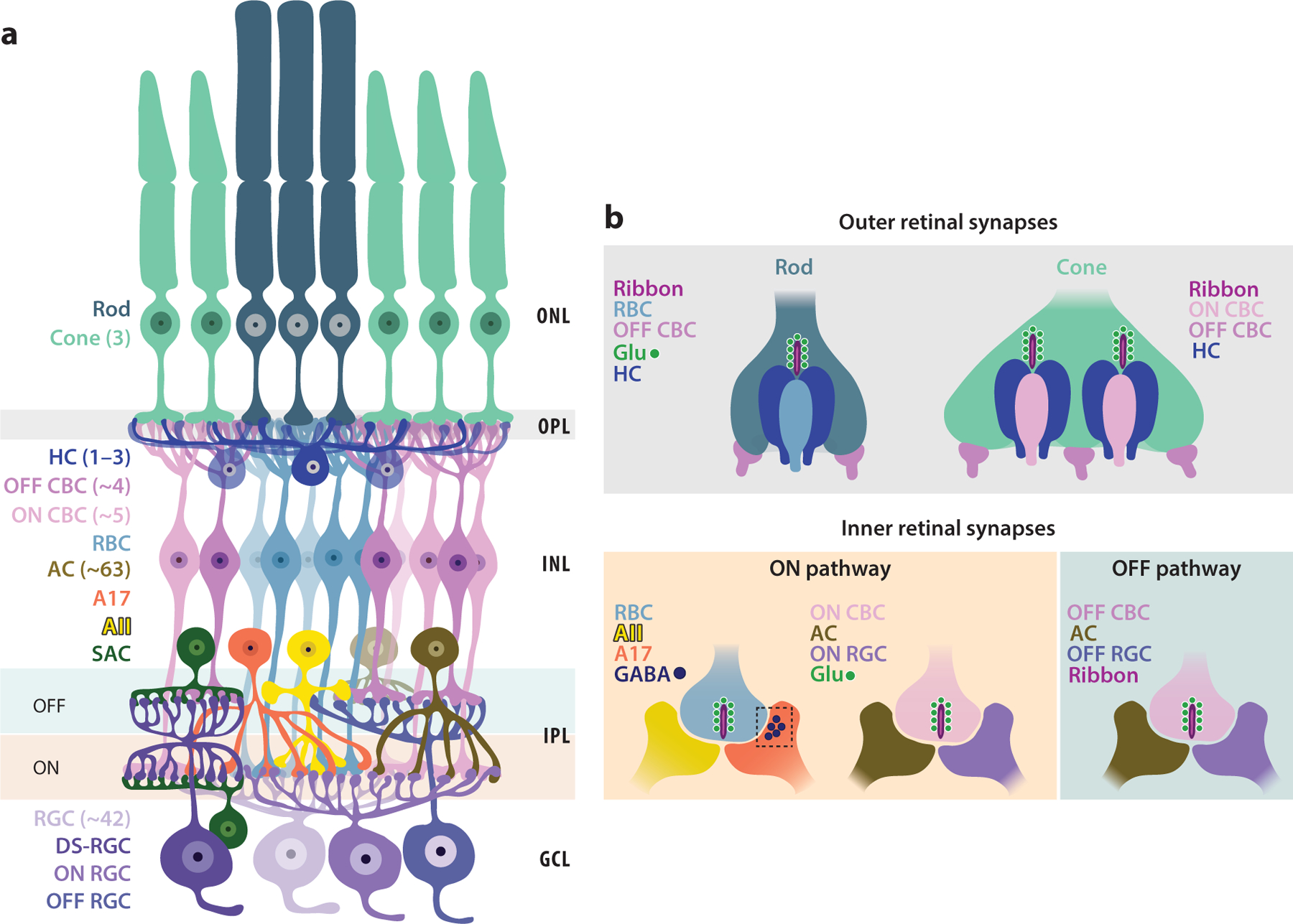
The mammalian retinal circuitry. (*a*) Schematic of the retina. Rod and cone photoreceptors reside in the outer nuclear layer (ONL) and synapse with bipolar cells (BCs) and horizontal cells (HCs) in the outer plexiform layer (OPL). BC and HC cell bodies are found in the inner nuclear layer (INL). The rod pathway relays dim-light or scotopic information via rod BCs (RBCs) that synapse onto AII and A17 amacrine cells (ACs). Bright-light or photopic information is conveyed via cone pathways, which have a direct line to output retinal ganglion cells (RGCs) via cone BCs (CBCs). RGC somas reside in the ganglion cell layer (GCL). AC somas lie in the INL and GCL (displaced ACs). The connectivity of neurons that depolarize to light increments or light decrements is restricted to the ON or OFF sublaminae of the IPL, respectively. Bistratified direction-selective RGCs (DS-RGCs) make synapses in both the ON and OFF sublaminae of the IPL and receive inhibition from starburst ACs (SACs) that is critical for responsivity to a preferred direction. The approximate numbers of subtypes per mammalian retinal neuron, as reported by Goetz et al. (2022), Hoon et al. (2014), Shekhar et al. (2016), Wassle (2004), and Yan et al. (2020), are indicated in parentheses. (*b*) The retina is equipped with specialized multipartner synapses. Glutamatergic ribbon synapses at photoreceptors and BC terminals provide excitatory inputs at the OPL and IPL, respectively. Inhibitory feedback is provided by HCs in the OPL and by ACs in the IPL. In the OPL, rod photoreceptor spherules make glutamatergic ribbon synaptic contacts with invaginating RBCs and HCs while making basal contacts with OFF CBCs. Similarly, cone pedicles make glutamatergic ribbon synaptic contacts with invaginating ON CBCs and HCs while making basal contacts with OFF CBCs. In the IPL, ACs provide inhibitory feedback for both ON and OFF pathways in the retina. In the rod pathway, RBCs make glutamatergic ribbon synapses onto AII and GABAergic A17 ACs. The A17 AC makes a reciprocal synapse back onto the RBC terminal and provides inhibitory feedback (*dotted box*). The ON and OFF CBC terminals establish ribbon synapses with ACs and type-specific (ON or OFF) RGCs.

The retinal circuit uses a multitude of synapses for processing visual information. In this review, we focus on the different chemical synapses used to connect retinal neuron types and the mechanisms that regulate synapse assembly and function. The retinal circuit is composed of diverse pathways that are each responsible for performing specific visual computations. Figure 1*a* displays the basic diagram and connectivity of retinal cell types; enclosed in parentheses are the numbers of subtypes that can exist for each retinal neuron. The primary glutamatergic pathway in the retina is established between photoreceptors and bipolar cells (BCs) and, thereafter, to output retinal ganglion cells (RGCs). Amacrine cell (AC) interneurons modulate information flow in the inner retina, whereas horizontal cells (HCs) regulate signaling in the outer retina (Figure 1). The diversity in the retinal circuit limits our ability to extrapolate the role of molecular mechanisms across cellular and synaptic types, as subtype-specific roles could regulate connectivity across pathways.

Of the two chemical synapse types employed by the retinal circuit, 1:1 conventional synapses are typically used by AC interneurons in the inner retina (Figure 1*b*), whereas ribbon synaptic connections are established in the outer and inner retinal synaptic layers by photoreceptors and BCs, respectively (Figure 1*b*). Retinal ribbon synapse connections are multipartner, with more than one postsynaptic partner receiving information from the presynaptic terminal (Hoon et al. 2014, Wassle 2004). Furthermore, the molecular composition of ribbon synapses is distinct from that of conventional synapses: Ribbon synapses have specialized proteins, such as ribeye, that constitute the ribbon (Heidelberger et al. 2005, Schmitz 2009), a specialization for facilitating high-throughput neurotransmitter (NT) release (Migdale et al. 2003, Schmitz 2009).

Outer and inner retinal ribbon synapses can also employ distinct molecular complements—the presynaptic protein bassoon, for example, is an integral anchoring component of photoreceptor ribbons but is missing from inner retinal ribbon synapses (Heidelberger et al. 2005). Instead, bassoon equips AC synapses in the inner retina (Brandstatter et al. 1999). Inner retinal conventional synapses and retinal ribbon synapses can also rely on distinct NTs: Retinal ribbon synapses are excitatory glutamatergic, but inner retinal conventional synapses can be either excitatory or inhibitory depending on the NT used (Hoon et al. 2014, Schmitz 2009, Wassle 2004). Together, this diverse cohort of synapse types ensures optimal signal flow and visual information processing through the retinal circuit.

Multipartner organizations at retinal ribbon synapses require a complex set of molecular and activity-dependent mechanisms to regulate precise organization and function. Indeed, as discussed below, several studies have found an extensive group of organizers for these specialized synapses. The primary literature reviewed in this article is from the mouse retina, a powerful mammalian model in which genetic tools, including loss-of-function [knockout (KO)] studies, enable interrogation of the localization and function of molecular organizers and assessment of circuit activity perturbations on synapse establishment. Our discussion of molecular and activity-dependent organizers is not an exhaustive list of all known regulatory factors. Instead, we highlight examples to showcase the diverse roles of these mechanisms. Where possible, we discuss functional roles as assayed by (*a*) global electroretinogram (ERG) recordings, composed of distinct waveforms that each represent the functionality of specific retinal neuronal types (Wachtmeister 1998); (*b*) population responses of RGCs through multielectrode-array (MEA) recordings; or (*c*) visual function from individual neurons, as probed through single-cell patch-clamp electrophysiology.

We broadly classify molecular organizers into three categories, but one molecule can belong to overlapping categories and can share structural and functional similarities with members across categories. The guiding principles that we reflect on regarding synapse formation in the mammalian retina are that (*a*) molecular organizers have distinct roles at retinal synapses, which can be noncanonical when compared to roles in other regions of the central nervous system; (*b*) the same family of organizers can have distinct roles at different retinal synapses; (*c*) one organizer can regulate different aspects of circuit connectivity by engaging in diverse interactions with multiple partners; (*d*) expression of molecular organizers can be regulated across development, with different isoforms equipping synapses at developing versus mature time points; and (*e*) activity perturbations or loss of cells can impact retinal synapse types and connections disparately.

## 2. ORGANIZING SYNAPSES IN THE OUTER RETINA

The primary synapse in the outer retina is the glutamatergic photoreceptor multipartner synapse, where the presynaptic photoreceptor terminals establish both invaginating and basal postsynaptic connections (Figure 1*b*). Photoreceptor synapses set up parallel output pathways to the inner retina: dim-light (rod) versus bright-light (cone) pathways and circuits that depolarize to increments of light (ON) versus decrements of light (OFF). The rod photoreceptor terminal or spherule makes invaginating ribbon synaptic contacts with the dendrites of rod BCs (RBCs) and axonal processes of HCs and basal contacts with dendrites of OFF cone BCs (CBCs). The cone photoreceptor terminal or pedicle makes invaginating ribbon synaptic contacts with dendrites of different ON CBC types and dendritic processes of HCs and basal synaptic contacts with dendrites of OFF CBCs. Compared to the rod photoreceptor synapse, the cone synapse has greater complexity; it establishes more than 10 parallel channels to the inner retina (Hoon et al. 2014). The mechanisms that establish these complex yet precise outer retinal connections are manifold.

### 2.1. Molecules Organizing Outer Retinal Synapses

Diverse molecular organizers have been found to regulate the establishment and function of outer retinal photoreceptor synapses. Whereas some organizers are directed at regulating HC contacts, others are directed at regulating BC dendritic arbors and connectivity with photoreceptors. Molecular organizers can also be differentially recruited across development. As reviewed below, our understanding of photoreceptor connectivity with ON BC dendrites has progressed further than our knowledge about organizers at OFF BC synapses. These two dendritic processes are equipped with distinct glutamate receptor (GluR) types; the GluR type determines their response polarity—metabotropic GluR 6 (mGluR6) is expressed at the dendritic tips of ON CBCs and RBCs, and ionotropic GluRs equip OFF BC dendrites.

#### 2.1.1. Adhesion proteins.

Adhesion proteins mediate interaction between retinal neurons. Adhesion proteins belonging to the immunoglobulin and leucine-rich repeat (LRR) family of organizers can regulate the arrangement of outer retinal neurites and the establishment and function of outer retinal synapses. Discussed in this section are five such groups of organizers.

##### 2.1.1.1. AMIGO.

**Figure 2:**
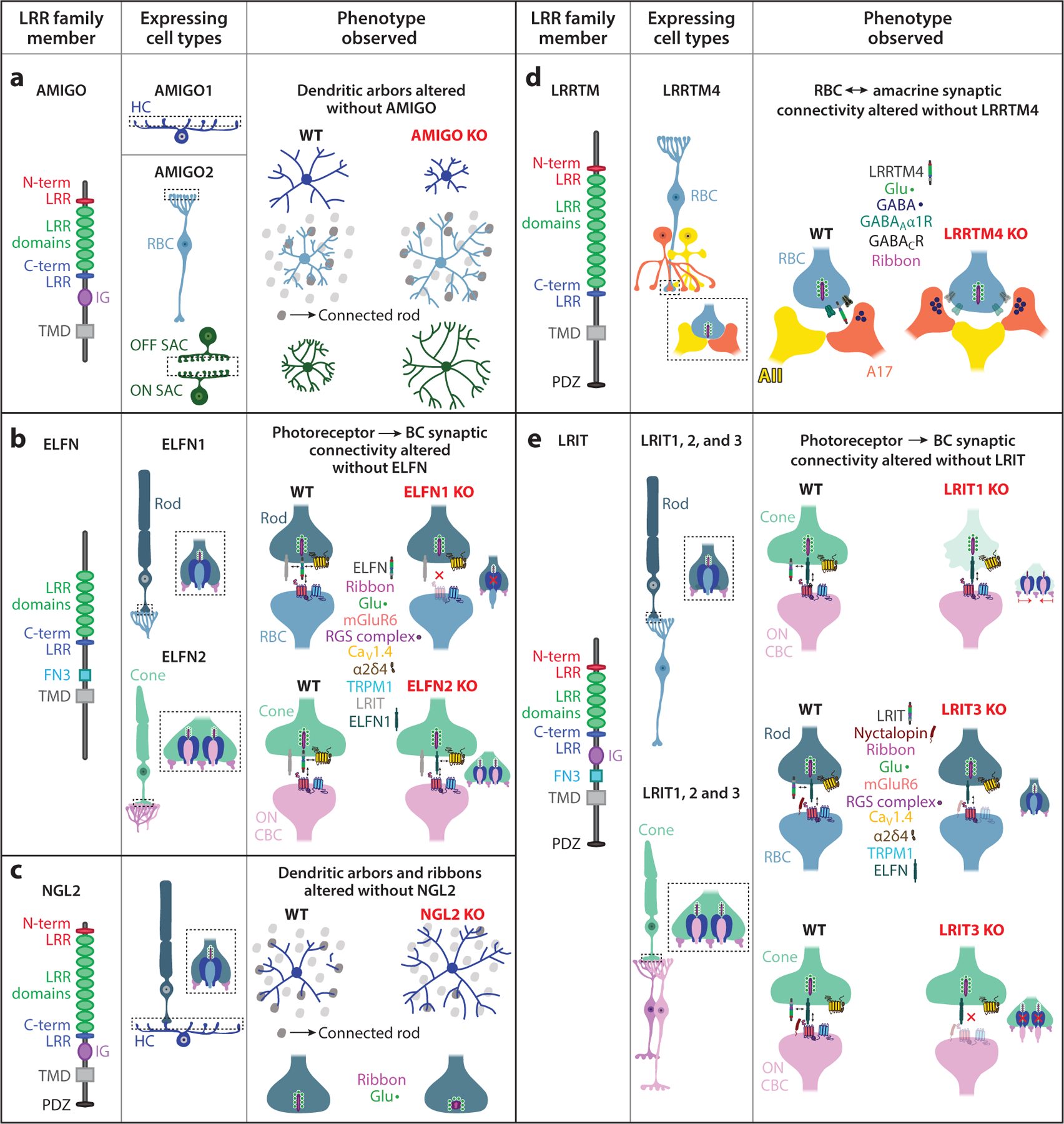
The role of leucine-rich repeat (LRR) proteins in regulating retinal synaptic connectivity. LRR proteins have distinct roles in establishing the molecular composition and connectivity of retinal synapses. Shown is a summary of the gross structural composition of select LRR proteins in the retina and the effects observed after their removal [comparison of knockout (KO) versus wild-type (WT) conditions]. (*a*) Amphoterin-induced gene and open reading frame (AMIGO) proteins have roles in shaping the arbors of neurons in both the outer and inner retina. AMIGO proteins have six LRR domains flanked by N-terminal (N-term) and C-terminal (C-term) LRR domains, an immunoglobulin (IG) domain, and a transmembrane domain (TMD) (Kajander et al. 2011, Kuja-Panula et al. 2003). In the outer retina, AMIGO1 is expressed by horizontal cells (HCs) and promotes HC axon growth. HC axons shrink in AMIGO1 KOs, but WT-like function is preserved (Soto et al. 2022). AMIGO2 is expressed in rod bipolar cells (RBCs) and in ON and OFF starburst amacrine cells (SACs). RBCs in AMIGO2 KOs expand their dendritic arbors and increase their synaptic connectivity with rod photoreceptors (Soto et al. 2019). SAC dendrites expand in the AMIGO2 KO but preserve their dendritic distribution to provide WT-like asymmetric inhibition to direction-selective retinal ganglion cells (DS-RGCs). (*b*) Extracellular LRR fibronectin type III domain containing (ELFN) proteins are expressed in both rod and cone photoreceptors, where they regulate synaptic connectivity. ELFNs contain several LRR domains, a C-terminal LRR domain, a fibronectin type 3 (FN3) domain, and a TMD (Dolan et al. 2007). (*Top*) ELFN1 is expressed in rod photoreceptors, where it interacts with Ca_V_1.4 via its α2δ4 subunit (Wang et al. 2017) and with another LRR domain protein, LRIT (Ueno et al. 2018). ELFN1 also interacts with postsynaptic metabotropic glutamate receptor 6 (mGluR6) at RBC dendrites. ELFN1 KOs have reduced expression of mGluR6 at RBC dendrites, and expression of several components of the regulators of G-protein signaling (RGS) complex (RGS7, RGS11, GPR179) is also reduced (Cao et al. 2015). Furthermore, rod photoreceptor terminals in ELFN1 KOs lack invaginating ON RBC dendrites (*side schematic*). (*Bottom*) ELFN2 is found in cone photoreceptors and interacts with presynaptic Ca_V_1.4 channels independently from their α2δ4 subunits and with mGluR6 at ON cone bipolar cell (CBC) dendrites (Cao et al. 2020). After ELFN2 KO, ELFN1 occupies cone terminals, although ELFN1 is not typically present in adult WT cones. No molecular or structural changes are observed at cone pedicles in ELFN2 KOs. (*c*) Netrin-G ligand 2 (NGL2) proteins contain nine LRRs flanked by N- and C-terminal LRRs, an IG domain, a TMD, and a PSD-95/Dlg/ZO-1 (PDZ)-binding motif (Seiradake et al. 2011). NGL2 is expressed in HCs, where it regulates axon growth. NGL2 KOs have overgrown HC axons and reduced synapses with rod photoreceptors (Soto et al. 2018). Moreover, the synaptic ribbons within rod terminals are misshapen and appear to be spherical or club-shaped in NGL2 KOs (Soto et al. 2013). (*d*) LRR transmembrane (LRRTM) proteins contain 10 LRR domains flanked by N- and C-terminal LRRs, a TMD, and a PDZ domain (Yamagata et al. 2018). LRRTM4 is expressed by RBCs, where it regulates GABA receptor clustering at axon terminals. GABA_A_ and GABA_C_ receptor clustering is reduced at LRRTM4 KO RBCs. Furthermore, RBC ribbon output sites across ACs are perturbed in the LRRTM4 KO, leading to erroneous synaptic assembly profiles, a reduction in the number of correct stereotypic AII–A17 dyads, and an increased number of synaptic vesicles at A17 boutons (Sinha et al. 2020). (*e*) LRR, immunoglobulin-like domain and transmembrane domain-containing (LRIT) proteins contain approximately five LRRs flanked with N- and C-terminal LRRs, an IG domain, an FN3 domain, and a TMD (Gomi et al. 2000, Ueno et al. 2018). LRITs 1 and 3 have PDZ domains, but LRIT2 does not. LRITs 1, 2, and 3 are all expressed in both rod and cone photoreceptors. In cone photoreceptors, LRITs 1 and 2 interact with mGluR6 at ON CBCs (Sarria et al. 2018, Ueno et al. 2018), and LRIT1/2 can form a complex with ELFN1 at the photoreceptor compartment (Ueno et al. 2018). LRIT3 interacts with nyctalopin at ON BC dendrites (Gregg et al. 2023; Hasan et al. 2019, 2020). LRIT1 KOs have shrunken and diffuse cone pedicles, but molecular composition at the cone→ON CBC synapses remains largely intact (Sarria et al. 2018, Ueno et al. 2018). LRIT3 KO rod photoreceptor synapses have reduced expression of transient receptor potential (TRP) M1 channel and nyctalopin at RBC dendrites, whereas LRIT3 KO cone photoreceptor synapses have reduced mGluR6 and RGS complex expression, in addition to loss of TRPM1 and nyctalopin (Hasan et al. 2020, Neuille et al. 2015). Moreover, ON CBC invaginations into cone photoreceptors are lost in the LRIT3 KO, whereas rod photoreceptor terminals remain unaffected. LRITs are shown in photoreceptors in this summary, but studies have reported that LRIT1 (Ueno et al. 2018) and LRIT3 (Hasan et al. 2020, Neuille et al. 2015) are also expressed by BCs. Structural representations for each LRR protein show the estimated domain numbers. Black bidirectional arrows represent a subset of molecular interactions. Phenotypes are depicted at the adult time point.

Members of the LRR family of proteins regulate the organization, connectivity, and function of photoreceptor synapses (Figure 2). AMIGO2, a member of the amphoterin-induced gene and open reading frame (AMIGO) family of LRR proteins, is expressed by RBCs and regulates their dendritic arbors. In the AMIGO2 KO, the dendritic arbors of RBCs expand and make more photoreceptor synaptic contacts (Soto et al. 2019). Another member of the AMIGO family, AMIGO1, is expressed by HCs (Soto et al. 2022). In mice with global AMIGO1 deletion or AMIGO1 deletion in individual HCs via viral infection, the HC axonal arbor size shrinks (Figure 2), leading to mislamination into the outer nuclear layer (ONL), but HC dendrites and soma distribution remain unchanged. Moreover, AMIGO1 deletion has no effect on the densities of HC axon tips or dendritic clusters, which invaginate rod spherules and cone pedicles, respectively, underscoring that AMIGO1 loss does not impact outer retinal synaptic density.

Although RBCs do not express AMIGO1 themselves, their dendrites shrink in parallel with HC axons in AMIGO1 KO mice, presumably due to compensatory mechanisms aimed at matching territories of synaptic partners. In vivo dim-light or scotopic ERG recordings that probe rod photoreceptor→RBC functional transmission in AMIGO1 KOs showed no effects in the *a*- or *b*-waves, which reflect rod and RBC responses, respectively. Although AMIGO2 is expressed in RBCs, it does not functionally compensate for the loss of AMIGO1 in the AMIGO1/2 double KO (Soto et al. 2022). MEA recordings from RGCs in AMIGO1 KOs showed conserved light responses, indicating that the territory matching between HCs and RBCs in AMIGO1 KO mice can stabilize retinal function. Whether ultrastructural changes arise within the tripartite rod photoreceptor synapses formed by HC axons and RBC dendrites in AMIGO1 KO retinas is not yet known, and it also remains unknown whether members of the AMIGO family could play similar roles at cone terminal synapses.

##### 2.1.1.2. ELFN.

Different isoforms of another LRR family of organizers, the extracellular LRR and fibronectin type III domain containing (ELFN) family of proteins, regulate photoreceptor synaptic connectivity (Figure 2). In contrast to their role at the postsynaptic compartment of hippocampal and cerebral cortex synapses (Matsunaga & Aruga 2021), ELFNs are expressed presynaptically at the photoreceptor terminal and interact with mGluR6 that is enriched at ON BC (i.e., at RBC and ON CBC) dendrites. ELFN proteins are thought to allosterically modulate mGluR6 function (Dunn et al. 2018). At rod synapses, ELFN1 regulates both ultrastructural and functional synapse formation with RBC dendrites. ELFN1 interacts in *trans* with mGluR6 expressed at RBC dendrites and in *cis* with Ca_V_1.4 channels (Figure 2) that regulate glutamate release from photoreceptor terminals (Williams et al. 2022). Global ELFN1 KOs have reduced rod→RBC contacts and decreased mGluR6 expression at RBC dendrites (Cao et al. 2015). In addition, the expression of several components of the regulators of G-protein signaling (RGS) complex (i.e., RGS7, RGS11, GPR179), which associate with and regulate mGluR6 signaling at ON BC dendrites, is reduced in ELFN1 KO RBCs.

Developmentally, ELFN1 and mGluR6 protein expression in the outer retina appear coincidentally when synaptogenesis peaks around postnatal day (P) 14. In ELFN1 KOs, the canonical ON RBC invaginations into rod spherules are lost, but the ON CBC invaginations remain intact at the cone pedicles, as revealed by single-section transmission electron microscopy (TEM). ERG recordings from ELFN1 KOs show no RBC-specific *b*-wave in response to dim-light flashes, but cone-driven or photopic light responses remain intact (Cao et al. 2015). Furthermore, ON and OFF CBCs showed robust responses to light flashes in ELFN1 KOs, as determined by single-cell patch-clamp recordings. ELFN1 is therefore required for the formation of rod→RBC synaptic connections.

At cone terminals, however, two different ELFN isoforms regulate the early and late stages of synaptic connectivity with ON CBC dendrites. ELFN1 is expressed early in development, with levels of ELFN1 downregulating at P11 in the rodent retina (Cao et al. 2020), which is after the initial establishment of the primary retinal glutamatergic pathway but prior to the onset of robust visually driven activity that occurs around eye opening (P14). ELFN2 expression in cones increases after P7, reaching adult or mature levels by three weeks of age. Thus, ELFN1 and ELFN2 protein expression is temporally separated during cone synaptogenesis. Notably, these two ELFN isoforms are coregulated at cones such that loss of one isoform triggers increased expression of the other (Cao et al. 2020).

Presynaptic photoreceptor interactions, not transsynaptic interactions with BC proteins, regulate ELFN2 expression: In leucine-rich repeat, immunoglobulin-like domain and transmembrane domain-containing protein (LRIT) 3 (a member of another class of LRR proteins localized at photoreceptor terminals) KO and Ca_V_1.4 KO mice models, the synaptic accumulation of ELFN2 is reduced at cone terminals, whereas in TRPM1 (a component of the postsynaptic BC mGluR6 signaling cascade) KO or upon the loss of different RGS proteins (i.e., RGS7 KO and RGS11 KO), ELFN2 synaptic targeting remains unaffected (Cao et al. 2020). Notably, ELFN2 KOs had no protein expression changes in key synaptic molecules, including mGluR6 and TRPM1, at ON BC dendrites.

In contrast to ELFN1 KO, ELFN2 KO yielded no retinal architectural changes. Furthermore, ERG recordings in ELFN2 KOs revealed no changes in wave components across light conditions. At cone synapses, though, loss of both ELFN isoforms triggers a downregulation of mGluR6 levels at ON CBCs and functional impairments but no ultrastructural misorganization of cone terminals (Cao et al. 2020). Ca_V_1.4 and its auxiliary subunit α2δ4 also regulate ELFN expression: Ca_V_1.4 KO or α2δ4 KO leads to reduced ELFN1 expression at rod terminals (Cao et al. 2015, Wang et al. 2017), underscoring the multifarious nature of the regulatory complex that orchestrates photoreceptor→BC connectivity.

##### 2.1.1.3. LRIT.

LRITs make up another class of LRR proteins that bear structural similarities to ELFN proteins and that regulate synapse organization and function in the outer retina. LRITs localized at both rod and cone terminals regulate photoreceptor connectivity (Hasan et al. 2019, Sarria et al. 2018, Ueno et al. 2018) (Figure 2). LRIT1 KOs have abnormal cone pedicle synapses that are smaller than control or wild-type (WT) retinas, whereas rod spherules remain undisturbed. Functional deficits are observed in the photopic ERGs of LRIT1 KO mice, indicating that the speed of cone photoreceptor→ON CBC transmission is delayed (Ueno et al. 2018). At the behavioral level, impairments in the optokinetic responses were observed in global LRIT1 KOs (Sarria et al. 2018, Ueno et al. 2018) but not in mice lacking LRIT1 specifically from ON BCs (Ueno et al. 2018), indicating that the reduction in visual acuity in response to moving objects is due to photoreceptor-specific abnormalities imposed by LRIT1 deficiency. Single-plane TEM analyses demonstrated no ultrastructural connectivity changes of photoreceptor synapses in LRIT1 KOs (Sarria et al. 2018, Ueno et al. 2018).

Loss of LRIT1 specifically from ON BCs does not impact cone synapse morphology or function, indicating that LRIT1 expression in photoreceptors but not BCs is necessary for accurate cone→BC synapse formation (Ueno et al. 2018). Additionally, deletion of mGluR6 or TRPM1 has no effect on LRIT1 expression, whereas removal of the Ca_V_1.4 channel (Ca_V_1.4 KO) or its α2δ4 accessory subunit (α2δ4 KO) at the photoreceptor compartment results in an enrichment of LRIT1, indicating that LRIT1 expression is dependent on photoreceptor activity (Sarria et al. 2018). LRIT2 is also expressed in photoreceptors and BCs but was determined to be dispensable for outer plexiform layer (OPL) synapse formation (Ueno et al. 2018). Indeed, LRIT1/2 double KOs and LRIT1 KOs display almost identical phenotypes. Interestingly, ELFN1 and LRIT1/2 coimmunoprecipitate, suggesting that LRITs could form complexes with ELFNs at the rod terminal. LRR proteins engaging in heteromeric complexes at photoreceptor terminals could thus work in a concerted manner to regulate synapse formation. Given the complex structure of photoreceptor terminals, the need for a multitude of molecular organizers is not surprising.

Another LRIT isoform, LRIT3, is additionally expressed by rod and cone photoreceptors (Gregg et al. 2023, Hasan et al. 2019) (Figure 2). In LRIT3 KOs, mGluR6 and GPR179 expression is preserved across rod appositions but is absent from cone synaptic appositions (Gregg et al. 2023, Hasan et al. 2019). Additionally, in LRIT3 KOs, TRPM1 is retained in the cell bodies of ON BCs and is not trafficked to the dendritic tips (Agosto et al. 2018, Gregg et al. 2023, Hasan et al. 2019, Neuille et al. 2015). RGC ON responses are abolished, and OFF responses are slowed, in LRIT3 KOs compared to WT (Neuille et al. 2017). Ultrastructural changes are also observed in LRIT3 KOs, as cone synapses have fewer invaginating ON CBC dendrites (Neuille et al. 2017). Interestingly, RBC single-cell recordings showed a loss of excitatory responses in LRIT3 KOs similar to those observed for TRPM1 KOs (Hasan et al. 2020). Thus, LRIT3 is necessary for appropriate photoreceptor synapse formation and function. LRIT3 is also expressed by BCs (Hasan et al. 2020, Neuille et al. 2015, Ueno et al. 2018) and could play additional roles in regulating information flow to the inner retina.

##### 2.1.1.4. Nyctalopin.

Another LRR protein critical for ON BC function is nyctalopin, which colocalizes with mGluR6 and TRPM1 channels at ON BC dendrites (Gregg et al. 2007, Pearring et al. 2011). Nyctalopin mutations cause a no *b*-wave (*nob*) ERG phenotype representing a lack of ON BC activation and are associated with congenital stationary night blindness (Gregg et al. 2003). Nyctalopin KOs lose TRPM1 in ON BC dendrites (Gregg et al. 2007, Hasan et al. 2020, Pearring et al. 2011), whereas global TRPM1 KOs have normal nyctalopin expression patterns but the same functional loss of excitatory inputs to ON BCs as the nyctalopin KOs (Hasan et al. 2020, Pearring et al. 2011). Like the LRIT3 KOs, nyctalopin KOs retain TRPM1 in ON BC cell bodies (Agosto et al. 2018, Hasan et al. 2019, Neuille et al. 2015, Pearring et al. 2011), suggesting that both molecules are required for the appropriate localization of TRPM1 channels to ON BC dendritic tips.

##### 2.1.1.5. NGL2 and SynCAM1.

HC axonal growth and synapse formation are also regulated by an LRR domain–containing synaptic cell-adhesion molecule, Netrin-G ligand 2 (NGL2) (Soto et al. 2018) (Figure 2). Deleting NGL2 from HCs causes overgrowth of HC axonal arbors and formation of fewer synapses. Interestingly, this deficit is observed both when the deletion is performed at the early stage and when it is performed at the late stages of circuit assembly. Viral restoration of NGL2 led to correction of this deficit even at the adult time point, underscoring the ability of NGL2 to operate as a synaptic regulator throughout the lifetime. NGL2 is expressed by HC axonal tips and interacts transsynaptically with Netrin-G2 expressed by rod photoreceptors. Global NGL2 KO thus leads to defects in rod photoreceptor ribbon assembly, in addition to the lateral expansion of HC axons, mislocalization of HC processes into the ONL, and formation of fewer synaptic connections with rod photoreceptors (Soto et al. 2013). Another adhesion molecule important for the assembly of rod synapses is the synaptic cell adhesion molecule 1 (SynCAM1), which is part of the immunoglobulin superfamily. Lack of SynCAM1 leads to formation of shorter rod photoreceptor ribbons; fewer triadic profiles constituting two HC and 1 RBC process within the rod terminal; proliferation of HC processes into the ONL; and functional deficits in rod transmission, as assayed by ERG recordings (Ribic et al. 2014).

#### 2.1.2. Synaptic proteins.

Proteins involved in the functioning of outer retinal synapses regulate OPL connectivity. These proteins can be associated with the presynaptic vesicle release compartment or the postsynaptic receptor compartment, or they can be part of the ribbon structure itself. Ribbon synapse proteins at photoreceptor terminals shape outer retinal circuit assembly and function. Lack of ribeye leads to reduced *b*-wave ERG amplitudes in dim but not bright-light conditions, indicating a deficiency in the rod photoreceptor→RBC synaptic transmission (Fairless et al. 2020). Photoreceptor terminals in this mutant also do not exhibit a luminance-dependent alteration in the protein clusters constituting the active zone (Dembla et al. 2020), a feature typically observed at WT photoreceptors. Another constituent of the photoreceptor active zone, bassoon, has been shown to be critical for the anchoring of photoreceptor ribbons and concomitant synaptic transmission (Dick et al. 2003). Notably, mutants with suppressed photoreceptor neurotransmitter release, such as the bassoon mutant, induce sprouting of postsynaptic BC and HC processes into the retinal ONL. In many of these cases, ectopic ONL synapse formation is also observed.

Other presynaptic proteins at the photoreceptor compartment also play regulatory roles for OPL synapses. An isoform of the presynaptic protein Neurexin regulates synapse formation for a subtype of cone photoreceptors. In short-wavelength (S)-cones lacking Neurexin 3, synaptic connections with S-cone CBCs are reduced (Kunze et al. 2023). Complexins make up another group of presynaptic proteins involved in regulating vesicular NT release, with Complexins 3 and 4 localized at retinal ribbon synapses (Reim et al. 2005). Complexin3/4 double KOs exhibit prominent impairment in the scotopic ERG *b*-wave amplitudes and latency, highlighting an inefficient rod photoreceptor→RBC transmission (Reim et al. 2009). Ultrastructural misorganization of rod photoreceptor ribbons (with club-shaped and free-floating profiles) are additionally observed in these mutants.

Ca_V_1.4 channels at photoreceptor terminals also direct photoreceptor connectivity. Interestingly, the conductance through the channel and the physical presence of the channel have distinct roles in the function and establishment of outer retinal synaptic contacts (Kerov et al. 2018, Maddox et al. 2020, Wang et al. 2017). In Ca_V_1.4 KOs, photoreceptor synaptic terminals are misshapen; lose molecular markers typically found in WT retinas, including ELFN1; and have immature, spherical ribbon profiles distinct from the elongated profiles observed in WT retinas (Cao et al. 2015, Liu et al. 2013). In Ca_V_1.4 knockin (KI) mutants, where Ca^2+^ influx through the channel is prevented without disrupting localization, rod ribbons similarly possess an immature, spherical shape (Maddox et al. 2020). KI retinas, however, conserve pre- and post-synaptic molecular elements that are completely lost in Ca_V_1.4 KOs (Cao et al. 2015, Maddox et al. 2020). Additionally, lamination of HCs and RBCs is drastically impaired in Ca_V_1.4 KOs but is less affected in the KI. Consequently, rod transmission is impaired in the KI but not lost entirely, as observed in Ca_V_1.4 KOs. Ultrastructural deficits are observed in Ca_V_1.4 KI retinas, as canonical BC and HC invaginations into rod photoreceptors are absent and replaced with basal contacts (Maddox et al. 2020). Furthermore, accuracy of photoreceptor synaptic configurations and selection of partners is disturbed in the KI, underscoring the importance of channel activity in regulating this aspect of synaptogenesis.

The auxiliary subunit for Ca_V_1.4, α2δ4, is also required for the functional establishment of photoreceptor synapses. In α2δ4 KOs, rod terminals retract into the ONL, with RBC dendrites sprouting past the OPL and into the ONL, resulting in OPL thinning (Kerov et al. 2018, Wang et al. 2017). This mislamination leads to an ultrastructural disarray in outer retinal synaptic organization, as α2δ4 KOs lose invaginating RBC and HC processes into rod spherules and possess shrunken cone pedicles, albeit with the same pedicle density (Kerov et al. 2018, Wang et al. 2017). α2δ4 KOs have decreased expression of photoreceptor synaptic proteins, with rods being impacted more than cones. Indeed, rod Ca^2+^ currents are reduced, and the voltage sensitivity and, thus, the biophysical properties of Ca_V_1.4 channels are altered in α2δ4 KOs (Kerov et al. 2018, Wang et al. 2017). These alterations lead to reduced rod→RBC transmission. Behavioral deficits are also observed in α2δ4 KOs, comparable to those of ELFN1, TRPM1, and mGluR6 KOs (Wang et al. 2017) but less severe than alterations observed in Ca_V_1.4 KOs (Kerov et al. 2018). Whereas rod→RBC transmission is completely absent in α2δ4 KOs, ON and OFF CBC pathways retain some activity (Wang et al. 2017). This speaks to potential plasticity processes that may act to preserve retinal functionality. To this point, heterozygous α2δ4 (α2δ4^+/−^) mutants conserve a WT-like function and ultrastructure, indicating that one allelic copy of α2δ4 is sufficient (Kerov et al. 2018). The mechanisms by which removal of α2δ4 has differential effects on rods versus cones are still unclear, but presynaptic interactions with photoreceptor-specific proteins could be regulatory. ELFN1 and α2δ4 interact and are codependent, as ELFN1 is lost from rod terminals in α2δ4 KOs (Cao et al. 2015, Wang et al. 2017). α2δ4 deletion does not, however, disturb ELFN2 expression at cone terminals (Cao et al. 2020), which could explain why cone synapses are more resilient to α2δ4 deletion. α2δ4 deletion also has disparate effects on the glutamate receptors equipping ON versus OFF BC dendrites—mGluR6 is reduced at the OPL, but GluR2 expression is preserved (Kerov et al. 2018).

As discussed in the previous section, postsynaptic proteins at the BC compartment also regulate OPL connectivity. Specifically, ON BC proteins related to mGluR6 signaling play critical roles in regulating synaptic transmission. To this end, RGS11, RGS9 anchor protein, and the short isoform of type 5 G-protein β (Gβ5) subunit form a complex and interact with mGluR6 at ON BC dendrites (Cao et al. 2009). RGS11 does not interact with the Gβ5 long isoform, which is more abundant in the retina, emphasizing a need for detailed biochemical studies to discern which elements belong to a particular molecular complex in specific retinal cell types. In *nob3* and *nob4* mice, where mGluR6 is not translated and not targeted to BC dendrites, respectively, expression of RGS complex proteins is impaired, indicating that, upon association with mGluR6, elements of the RGS complex are recruited to ON BC dendrites. Furthermore, single-plane TEM of *nob4* retinas showed that ON BC dendrites failed to invaginate rod terminals at the OPL, indicating a role for mGluR6 and/or RGS complex proteins in synapse formation (Cao et al. 2009). The roles of the OFF BC proteins and the interaction between ON and OFF signaling pathways for regulating OPL connectivity have remained comparatively understudied. Future work in this area could reveal OFF CBC–specific molecular organizers and cues that could correlate the functional establishment of ON–OFF retinal circuits.

#### 2.1.3. Extracellular and cytoskeletal-associated proteins.

Proteins that bridge the extracellular and cytoskeletal compartment also regulate OPL synapses. Two members of this protein family, dystroglycan and dystrophin, are localized on the photoreceptor plasma membrane (Schmitz & Drenckhahn 1997) and interact with Pikachurin, an extracellular matrix (ECM)-like protein that is localized to the synaptic cleft of cone pedicles and rod spherules (Sato et al. 2008). This interaction is dependent on the expression and post-translational maturation of dystroglycan (Hu et al. 2011, Kanagawa et al. 2010, Orlandi et al. 2018, Patil et al. 2023). Pikachurin also forms a complex with the orphan receptor GPR179 at ON BC dendrites (Orlandi et al. 2018). However, in the GPR179 loss-of-function mutant, Pikachurin expression is normal, and there are no defects in the localization of mGluR6 or TRPM1 at ON BC dendrites. Ultrastructural analysis of Pikachurin KOs revealed a loss of canonical BC invaginations at rod and cone photoreceptor synapses, although the BC terminals appeared to stay in close proximity to photoreceptor terminals. Pikachurin KOs also have reduced dystroglycan and RGS complex expression levels, but normal ELFN1, mGluR6, and TRPM1 expression (Omori et al. 2012, Orlandi et al. 2018, Sato et al. 2008).

The ERGs of Pikachurin KOs show functional abnormalities representative of impaired transmission from both rod and cone photoreceptors (Sato et al. 2008). Like the Pikachurin KO, the dystroglycan KO shows impaired BC invaginations into photoreceptor terminals and ERG deficits across light levels but normal mGluR6 expression at ON BC dendrites. Thus, loss of Pikachurin or dystroglycan reduces sensitivity of the photoreceptor→ON BC synapse, potentially owing to the loss of ON BC synaptic invaginations (Omori et al. 2012, Orlandi et al. 2018, Sato et al. 2008). Pikachurin expression is decreased in photoreceptor-specific dystroglycan KOs, which also lose ON BC invaginations into photoreceptor terminals (Omori et al. 2012, Orlandi et al. 2018). Pikachurin requires dystrophin for its localization, as its expression is reduced in mouse models with a partial functional loss of dystrophins (Orlandi et al. 2018). Furthermore, although Pikachurin is essential for the proper localization of dystroglycan at photoreceptor synapses and vice versa, dystrophin expression is not affected in the photoreceptor-specific dystroglycan KO (Omori et al. 2012), indicating that distinct molecular cues recruit these proteins to photoreceptor terminals.

Members of the 4.1 family of membrane cytoskeletal proteins are also regulators of OPL synaptic connectivity. 4.1G specifically is enriched at rod photoreceptors, and a lack of 4.1G leads to ectopic rod photoreceptor synapse formation in the ONL, with concomitant visual function disturbances (Sanuki et al. 2015). Whether 4.1 protein isoforms play similar roles at cone photoreceptor synapses remains to be determined, but it is clear that a complex array of extracellular and cytoskeletal-associated proteins regulate OPL synapse formation and function.

### 2.2. Cellular Mechanisms Organizing Outer Retinal Synapses

Activity-dependent mechanisms regulate connectivity at some synapses in the outer retina in part by altering synaptic protein expression. Preventing glutamate release from photoreceptors by selectively expressing tetanus toxin (TeNT), which abolishes vesicular fusion, results in disrupted photoreceptor→ON BC connectivity (Cao et al. 2015). No ELFN1 was identified at the photoreceptor terminals, nor was mGluR6 present at the tips of ON BC dendrites in TeNT retinas, implying that presynaptic release of glutamate regulates localization of these OPL proteins (Cao et al. 2015). Presynaptic targeting of ELFN1 was also lost in Ca_V_1.4 KO mice, which are deficient in photoreceptor glutamate release. Conversely, however, lack of ELFN1 does not impair Ca_V_1.4 expression or localization of synaptic ribbons at rod terminals (Cao et al. 2015). Thus, recruitment of ELFN1 and the consequent synapse formation require functional presynaptic vesicular release from photoreceptors and the physical presence of Ca_V_1.4 channels. However, additional studies using nonconducting Ca_V_1.4 channel mutants might provide valuable insight into whether conductance through Ca_V_1.4 channels and/or the physical presence of Ca_V_1.4 are necessary for the recruitment of ELFN1 and appropriate photoreceptor synapse formation.

Dark rearing can also influence the establishment and function of outer retinal synapses. Cone photoreceptor→ON CBC transmission is reduced in dark-reared animals, as shown by the smaller *b*-wave ERG responses and reduced clustering of mGluR6 at the dendritic tips of dark-reared ON CBCs (Dunn et al. 2013). mGluR6 clustering at RBC dendrites, however, remains unaltered in dark-rearing conditions, as do the ERG profiles representing the rod photoreceptor→RBC transmission (Dunn et al. 2013). Dark-rearing also regulates the orientation of photoreceptor inner segments (Chai et al. 2020) and the positioning of cone photoreceptors (Tufford et al. 2018). Interestingly, the mechanisms regulating the positioning of cone photoreceptors seem to be driven by intrinsically photosensitive RGCs (ipRGCs), as silencing ipRGCs produces errors in cone positioning similar to those produced by dark-rearing (Tufford et al. 2018). Thus, activity perturbations imposed by sensory deprivation can shape the organization and connectivity of outer retinal neurons.

Loss of cellular populations can lead to alterations in OPL synaptic connectivity and retinal function. Loss of HCs can impact rod→RBC connections, as RBC dendrites do not enter rod terminals upon HC ablation (Nemitz et al. 2019). The formation of invaginating ON CBC processes into cone photoreceptors also seems to be determined by HCs, as cone terminals lack ON CBC contacts when HCs are removed from the circuit during development (Nemitz et al. 2021). At the RGC level, loss of HCs alters the receptive field structure and response properties of RGCs (Chaya et al. 2017).

Photoreceptor loss can also induce alterations in connectivity. Site-specific loss of mGluR6 expression is observed at the dendritic arbors of ON CBCs that directly appose ablated cone photoreceptors (Care et al. 2019, Dunn 2015). This loss of glutamate receptors occurs even after pharmacological occupation of mGluR6 receptors, suggesting that physical loss of the presynaptic contacts, but not mGluR6 activation, drives this reduction. The CBC dendritic tips, additionally, exhibit minor changes in their morphology following cone pedicle ablation (Dunn 2015).

Controlled removal of 50% of cone photoreceptors, however, leads to BC dendritic remodeling, together with reduced mGluR6 expression at ON CBC dendrites (Care et al. 2019). Ablation of 50% of cones also leads to other structural rearrangements at the OPL: Remaining cone pedicles increase their size, and BC dendrites rewire their connections (Care et al. 2019, Shen et al. 2020). This retinal plasticity differs between BC subtypes and declines steeply with age (Shen et al. 2020).

Synaptic remodeling events seem to be geared to preserve retinal function after photoreceptor loss. When cone photoreceptor density is reduced by 50%, inner retinal BC→RGC synaptic function is largely conserved, and the primary output ganglion cells of the ON-pathway, the ONαRGCs, demonstrate a widening of their receptive fields to compensate for cone loss while slowing their response kinetics (Care et al. 2019). After 50% rod loss, the ONαRGCs exhibit a partial recovery of rod-mediated light responses and increase their cone-mediated responses (Care et al. 2020), indicating the recruitment of compensatory mechanisms. ONαRGCs also maintain their intrinsic excitability and identity after 50% ablation of either photoreceptor type (Care et al. 2019, 2020). The excitatory and inhibitory synaptic inputs received by the ONαRGCs after photoreceptor ablation are, however, altered (Care et al. 2019, 2020). Even after severe rod degeneration in a mouse model of advanced retinal degeneration, cones remain responsive to light, and some cone light-level responses are retained by downstream BCs and RGCs (Ellis et al. 2023). Altogether, these studies indicate that, although the extent of excitation and inhibition transmitted to the inner retina is altered due to photoreceptor loss, the net proportion of synaptic inputs to RGCs is largely preserved due to compensatory mechanisms. Special consideration, however, must be taken to distinguish cell-specific compensatory mechanisms from circuit-level compensatory properties.

At the postsynaptic OPL compartment, alteration of the BC population can spur changes in connectivity. RBC ablation alters the dendritic profile of the remaining RBCs and their connectivity with rods (Johnson et al. 2017). Specifically, WT RBCs frequently connect with a single rod via multiple postsynaptic densities (PSDs). Such an organization could promote a stronger rod→RBC synaptic connection compared to when a single PSD underlies this contact. When the RBC population is depleted by approximately 50–90%, the remaining RBCs expand their dendritic arbors and do not demonstrate multi-PSD contacts with rods, using single-PSD contacts instead. These mechanisms ensure that the dim-light RBC-mediated response profiles of RGCs are largely conserved even after approximately 50% RBC reduction (Johnson et al. 2017), highlighting homeostatic plasticity mechanisms that are geared toward conserving circuit function (for a review on retinal homeostatic plasticity, see Fitzpatrick & Kerschensteiner 2023). Interestingly, phototransduction does not regulate the formation of multi-PSD connections or the expansion of RBC dendritic profiles, as these connectivity parameters remain invariant in mutants with impaired rod phototransduction.

## 3. ORGANIZING SYNAPSES IN THE INNER RETINA

Inner retinal synapses can be broadly categorized into ribbon synaptic connections at BC terminals and conventional GABAergic and glycinergic inhibitory synapses made by ACs onto other ACs, RGCs, or BC terminals. GABA receptors and glycine receptors (GlyRs) are localized at distinct inner retinal synapses (Wassle et al. 1998). However, recent studies have identified a mixed GABA–glycine inhibitory synapse type across the dendritic arbors of the ONαRGC (Sawant et al. 2021). RBC terminals form dyadic connections with two AC types—the AII, which carries RBC signals to CBC output pathways, and the A17 GABAergic AC, which provides feedback inhibition onto the RBC terminal. AC→BC terminal synaptic connectivity is a motif called presynaptic inhibition that regulates NT release from BC terminals. CBC terminals form dyadic connections with an AC and an RGC partner, with pathway-specific connections being established (ON CBC→ON RGC and OFF CBC→OFF RGC) (Figure 1).

Retinal inhibitory synapses make up a diverse synapse population with specific receptor types equipping largely nonoverlapping synaptic subsets. Unique to the retinal circuit, distinct GABA_A_ synapses can be identified by the α-subunit incorporated within the receptor pentamer (Wassle et al. 1998). As such, three different types of GABA_A_ synapses with α1-subunit (GABA_A_α1)-, α2-subunit (GABA_A_α2)-, or α3-subunit (GABA_A_α3)-containing receptors play key roles in regulating information processing in the inner retina. Similarly, GlyR synaptic subsets can be typified by the incorporated α-subunit (α1–4) (Wassle et al. 1998). Diversity in inner retinal synaptic connectivity is also fueled by the different AC and RGC subtypes (Goetz et al. 2022, Yan et al. 2020).

### 3.1. Molecules Organizing Inner Retinal Synapses

Members of the LRR family play key roles in organizing inner retinal synapses. Additionally, synaptic proteins regulate the assembly and function of inner retinal circuits. Notably, members of the cadherin and semaphorin-plexin groups of adhesion proteins direct lamination of AC, BCs, and RGCs (for reviews and further reading, see Hamilton et al. 2021; Hoon et al. 2014; Matsuoka et al. 2011a,b), thereby organizing neuronal processes in the inner retina. In general, our knowledge of the organizers at inner retinal synapses is limited compared to our understanding of the processes underlying connectivity at the OPL. This is in part due to the extreme AC and RGC diversity (>40 types in each category), which can result in a multitude of connections.

#### 3.1.1. Adhesion proteins.

Similar to the outer retina, adhesion proteins belonging to the LRR family can organize neurite arrangement and synaptic connectivity in the inner retina. Additionally, members of the cadherin superfamily can also regulate inner retinal synaptic organization. This section discusses three examples of such molecular organizers.

##### 3.1.1.1. LRRTM.

LRR-domain proteins have been found to organize inner retinal synapses. LRR transmembrane protein 4 (LRRTM4), a member of the LRRTM family of postsynaptic organizers, has been found to regulate the clustering of receptors at GABAergic synapses across RBC terminals, thereby regulating NT release from RBC terminals and dim-light RGC output (Sinha et al. 2020). Lack of LRRTM4 causes reduced clustering of GABA_A_ and GABA_C_ receptors and a concomitant reduction in the extent of presynaptic inhibition received by RBC terminals (Figure 2). LRRTM4’s role at GABAergic synapses of the inner retina is unexpected given that, in the hippocampus and cortex, LRRTM4 regulates AMPA receptor clustering at glutamatergic synapses (de Wit et al. 2013, Siddiqui et al. 2013). Retinal LRRTM4 also regulates the ultrastructural organization of AC contacts onto RBC terminals and, by regulating the level of presynaptic inhibition, instructs the assembly of RBC ribbon sites at stereotypic AII–A17 dyads (Sinha et al. 2020).

Whether other members of the LRRTM family work in concert to organize and support retinal synapses remains to be determined, as does the synaptic interactions that LRRTM proteins engage in to establish inner retinal synapses. In this regard, both *cis* interactions at the same synaptic compartment and *trans* interactions with partners across the synaptic cleft would be informative in revealing the molecular basis of retinal synaptic assembly and function.

##### 3.1.1.2. AMIGO.

The other LRR-domain protein that regulates connectivity in the inner retina is AMIGO2 (Soto et al. 2019). AMIGO2 is expressed by starburst amacrine cells (SACs) (Figure 2) that are central to the retinal circuit that processes direction-selective (DS) visual information. In global AMIGO2 KOs, the dendritic arbors of SACs expand, whereas SAC density remains unchanged, as does the arrangement of ON and OFF SAC somas. Additionally, SACs maintain their branching and subcellular compartmentalization patterns in AMIGO2 KOs, and the dendritic distribution of AMIGO2-KO SACs is scaled proportionately to that of their WT counterparts to maintain appropriate self-avoidance and input–output division properties (Soto et al. 2019). SACs underlie DS-computations by providing asymmetric inhibition to DS-RGCs (Morrie & Feller 2016). This functional property was largely preserved in AMIGO2 KOs, as shown by paired patch-clamp recordings from SACs and DS-RGCs. Given that SAC density remains constant as arbors expand, dendrite coverage is higher in AMIGO2 KOs (Soto et al. 2019). This change in retinal architecture corresponds to an enhanced direction selectivity of mutant DS-RGCs compared to WT.

##### 3.1.1.3. Fat3.

A member of the atypical cadherin protein family, Fat3, regulates AC synapse formation. Membrane-bound Fat3 protein is enriched in the retinal inner synaptic or inner plexiform layer (IPL) throughout murine development (Nagae et al. 2007). Fat3 transcripts are expressed in the inner nuclear layer (INL), where ACs reside, and at the ganglion cell layer (GCL), which contains the cell bodies of both RGCs and displaced ACs. Fat3 protein is enriched at AC dendrites early in IPL development and is maintained throughout maturation. Global or AC-specific KOs of Fat3 have demonstrated several roles for Fat3 in retinal neuron migration and synapse formation. Fat3 affects AC morphology, restricts AC dendrite numbers, and regulates AC distribution (Deans et al. 2011, Krol et al. 2016). Interestingly, ACs lacking Fat3 develop extra dendritic arbors that project away from the IPL and migrate into the INL and GCL in excess numbers, thereby generating two additional synaptic or plexiform layers that are typically not present in WTs. These aberrant layers contain synaptic contacts, as synaptic proteins and partners are localized to these plexuses. Thus, Fat3 acts as a molecular regulator for inner retinal synaptic lamina development. Additionally, time-lapse imaging revealed that in the absence of Fat3, ACs are less directed toward the IPL during both migration and retraction (Krol et al. 2016). Notably, different motifs within the cytosolic domain of Fat3 are required for migration, neurite retraction, and synapse localization of ACs via multiple cytoskeletal effectors (Aviles et al. 2022), underscoring the engagement of specific molecular interactions that guide distinct aspects of inner retinal synaptogenesis.

#### 3.1.2. Synaptic proteins.

As detailed in the previous section, the LRRTM family of postsynaptic organizing proteins play a crucial role in regulating the establishment of inner retinal GABAergic synapses. Other postsynaptic adhesion proteins also regulate circuit assembly and function in the inner retina. Members of the Neuroligin family regulate receptor clustering and functioning of inner retinal inhibitory synapse types. Interestingly, different Neuroligin isoforms direct the organization of distinct retinal synaptic subsets: Neuroligin-2 KO leads to loss of GABA_A_α3 receptor clustering (Hoon et al. 2009), Neuroligin-3 KO leads to loss of receptor clustering at GABA_A_α2 synapses (Hoon et al. 2017), and Neuroligin-4 KO leads to reduced GlyRα1 receptor clustering (Hoon et al. 2011). Each Neuroligin-isoform KO also demonstrates functional impairments due to the impacted inhibitory synaptic subset. Notably, Neuroligins have not yet been reported to play a regulatory role at retinal ribbon synapses.

Other elements of the postsynaptic compartment also play regulatory roles at the IPL. The inhibitory postsynaptic scaffolding protein gephyrin is important for receptor clustering at both GABAergic and glycinergic inner retinal synapses (Fischer et al. 2000). Collybistin, a regulator and interaction partner of gephyrin (Papadopoulos & Soykan 2011), shows a preferential association with GABA_A_α2 synapses in the inner retina (Saiepour et al. 2010). The consequences of a lack of collybistin for the organization and function of retinal synapses, however, have not yet been determined.

GABA_A_α3 receptors themselves can serve as molecular organizers for IPL inhibitory synapses. At BC terminals, GABA_A_α3 is expressed before eye opening, and its expression is consequently downregulated (Sinha et al. 2021). After eye opening, GABA_A_α1 receptors are instead enriched at BC terminals. Early GABA_A_α3 expression, however, is critical for enabling the clustering of GABA_A_α1 receptors and for the establishment of functional GABA_A_ synapses at BC terminals (Sinha et al. 2021).

GABA_A_α3 also operates as a molecular organizer at RGC inhibitory synapses. ONαRGCs that respond to light increments and encode a wide range of luminosities express three inhibitory synapse types across their dendritic arbors: GABA_A_α3 receptor synapses, GlyRα1 receptor synapses, and synapses with mixed GABA_A_α3-GlyRα1 receptors (Sawant et al. 2021). Lack of GABA_A_α3 causes reduced clustering of GlyRα1 across ONαRGC dendrites, with the proportion of loss being similar to the mixed GABA_A_α3-GlyRα1 synaptic receptor fraction. Thus, at ONαRGC inhibitory synapses, early GABA_A_α3 recruits GlyRα1. The functional consequences of this redistribution of inhibitory receptor synapses in the GABA_A_α3 KO ONαRGC remains undetermined but may provide insights into the mechanisms that regulate inner retinal inhibition.

In the DS circuit, a distinct subtype of GABA_A_ receptors, GABA_A_α2, regulates synapse formation and circuit function (Auferkorte et al. 2012). Additionally, cholinergic receptors regulate DS-RGC function, as global β2-nicotinic acetylcholine receptor (β2-nAChR) KO reduces horizontal tuning in a fraction of both ON-OFF RGCs and ON DS-RGCs (Tiriac et al. 2022). Cholinergic transmission also sustains the function of SACs and is the basis of the early stage of spontaneous retinal activity (i.e., waves) (Ford & Feller 2012, Voufo et al. 2023). SACs release excitatory acetylcholine that activates nAChRs in both DS-RGCs and SACs. SAC-specific β2-nAChR KOs have truncated wave propagation and reduced wave size (Xu et al. 2016). In choline acetyltransferase (ChAT) KO mice, where most of the retina is devoid of cholinergic transmission, the morphology, lamination, and synapse sizes of SACs are, however, comparable to those of WT. Interestingly, although waves initially fail to propagate in the ChAT KO, they re-emerge through compensatory signaling mechanisms via the gap-junctional network (Stacy et al. 2005). The impact of these manipulations of cholinergic signaling on synaptic structures remains to be determined.

On the presynaptic compartment, lack of ribeye does not seem to perturb synapse formation or overall function of RGCs. In the ribeye KO, ONαRGCs had unperturbed excitatory synapse density across developmental time points, and synaptic dynamics at P11 remained comparable to WT. Light response properties of these RGCs also remained quite similar to WT, albeit with reduced frequency and contrast sensitivity (Okawa et al. 2019). Ribeye KO mice, however, do exhibit supernormal ERG oscillatory potentials, which are reflective of AC-mediated inhibitory feedback loops, underscoring that inner retinal circuit activity is altered in this KO (Fairless et al. 2020). Complexins make up another group of presynaptic proteins that regulate inner retinal synapse function. In particular, lack of Complexin 3 impairs fast RBC→AII signal transmission, impacting rod- but not cone-mediated signaling through RGCs (Mortensen et al. 2016).

#### 3.1.3. Extracellular and cytoskeletal associated proteins.

The role of ECM organizers for inner retinal synapse connectivity and visual function has remained understudied. These organizers, though, do regulate retinal function, as KO of four ECM molecules leads to impaired ERG responses and loss of AC types in the inner retina (Reinhard et al. 2023). The laminin class of glyco-proteins can regulate development of dopaminergic AC types (Denes et al. 2007), and dystroglycan regulates inner retinal circuit formation, as stratification of AC and RGC types is disrupted in the dystroglycan KO (Clements et al. 2017). It is likely that the 4.1 family of proteins, in addition to regulating OPL synaptic connectivity and function, could play additional roles in regulating inner retinal synapses. Indeed, this family of organizers is expressed by inner retinal neurons with varying patterns in developing versus mature retina (Sanuki et al. 2015). Additionally, molecules belonging to the cell-surface heparan sulfate proteoglycan (HSPG) family, such as glypican, are expressed by RGCs (Karthikeyan et al. 1994). N-syndecan is another HSPG molecule expressed by ACs and RGCs (Inatani & Tanihara 2002). Our understanding of the role of HSPGs in regulating synapses is expanding (Condomitti & de Wit 2018), and future studies will likely reveal new roles for this group of organizers at retinal synapses.

### 3.2. Cellular Mechanisms Organizing Inner Retinal Synapses

Loss of inner retinal cell types imposes alterations in synaptic connectivity, as partner selection is regulated by cell density and the availability of preferred partners. Ablation of ON BCs by diphtheria toxin expression results in increased RGC synaptic connectivity with the remaining BCs (Okawa et al. 2014). Removal of the preferred ON BC partner also prompts the ONαRGC to erroneously extend into the OFF lamina and establish new functional synaptic connections with OFF BCs (Okawa et al. 2014). Interestingly, the selected OFF BC type in this scenario shares some molecular similarity with the preferred ON BC type, underscoring that molecular matching programs may be operational. BC→RGC connectivity is also regulated by homeostatic plasticity mechanisms. Type-6 ON BCs are the preferred partners of ONαRGCs (Schwartz et al. 2012), but when these BC types are selectively removed from the circuit during development, the ONαRGC can recruit new partners and increase synaptic connections to preserve functional responses (Tien et al. 2017).

Another mechanism to consider during the establishment of inner retinal synapses is a hierarchy in partner selection, which is presumably genetically preprogrammed. When the RBC population is selectively reduced, the A17 AC directs synaptic connections with CBCs instead (Zhang et al. 2022). As inner retinal ACs or RGCs are not selected in this scenario, A17 ACs display cell-type specificity in selecting BCs as postsynaptic partners. The cues that direct this specificity remain to be determined but are a fascinating avenue for future exploration.

In the *bipolarless* mouse, when the preferred AII AC partners (BCs) are lost, AII ACs maintain the strength of non-BC synaptic connections even in a scenario that would allow for increased contacts with these non-BC partners (Gamlin et al. 2020). This indicates that cell-intrinsic mechanisms could regulate output connectivity and selection of partners and that some inner retinal neurons would rather not establish synaptic connections when their preferred partner population is missing. Molecular matching across BC versus AC or RGC populations could determine the specificity in the synaptic connections made by the AII AC. Taken together, these results indicate that crosstalk among varied cellular mechanisms underlies synaptic connectivity and function in the inner retina, and these divergent mechanisms regulating inner retinal synaptic connectivity could explain the variation in pathophysiology observed during retinal diseases: Neuron- and pathway-specific measures can be selectively recruited to compensate for the lost synaptic fraction in some scenarios and would not be employed in other cases.

The balance between excitation and inhibition in the network is another mechanism guiding inner retinal synapse formation. Blocking glutamatergic NT release from ON BC terminals by expression of TeNT reduces synaptic connectivity between ON BCs and RGCs, resulting in reduced synapse formation and fewer BC active zones that recruit multiple presynaptic ribbons (Kerschensteiner et al. 2009). Matching between ON BCs and ON RGCs, however, remains unperturbed in these conditions (Kerschensteiner et al. 2009). This study also confirmed the independent formation of ON versus OFF lamina synapses, as ON-OFF RGCs in TeNT retinas selectively reduced synaptic connections with ON BCs without altering connectivity with OFF BCs (Kerschensteiner et al. 2009). This activity-dependent regulation of ON BC→RGC connectivity seems to be operational on a cell-by-cell basis, as individual BCs that are deficient in neurotransmission can reduce the number of RGC synapses (Okawa et al. 2014).

**Figure 3:**
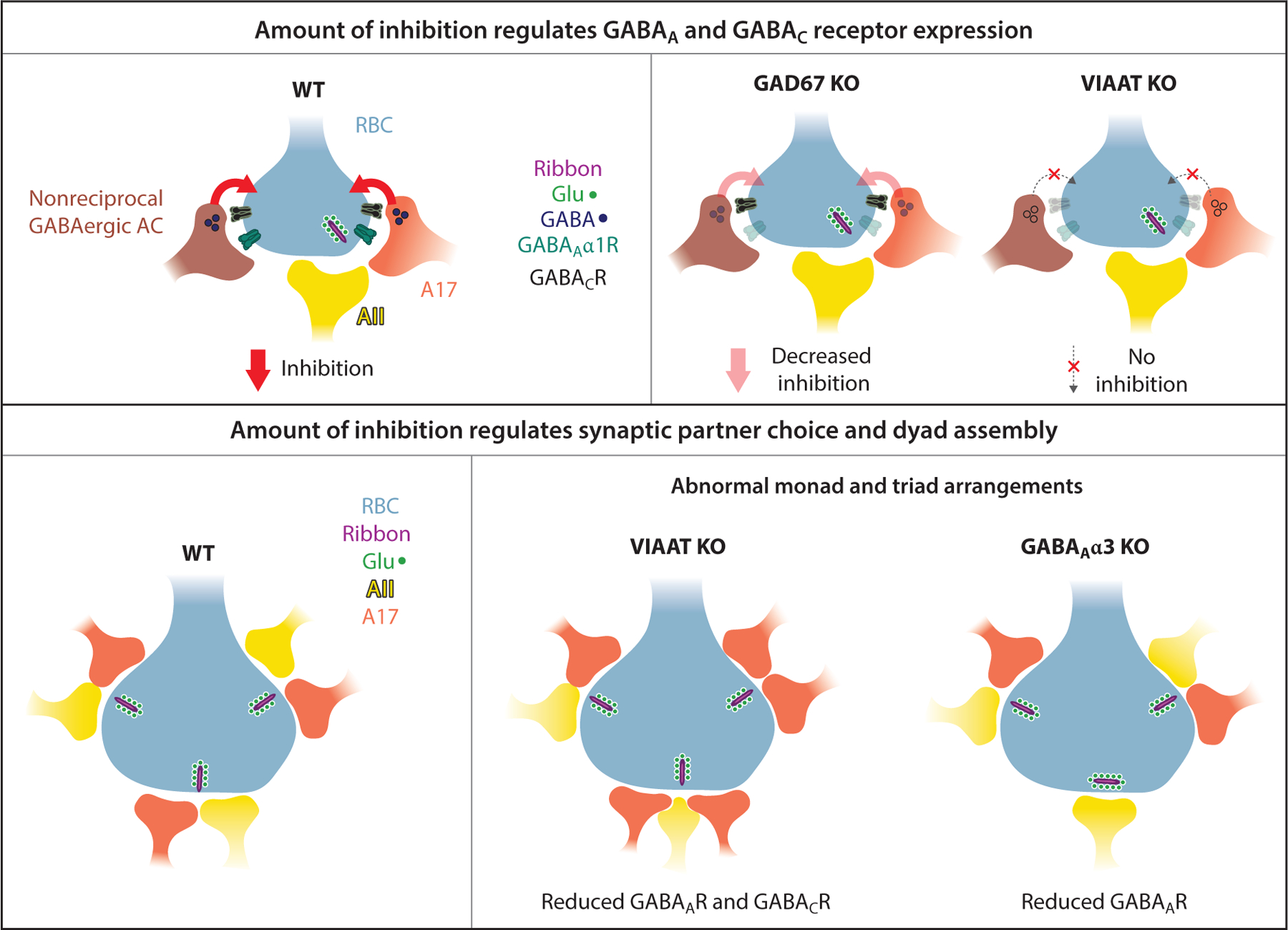
Activity-dependent mechanisms shape the establishment of presynaptic inhibitory circuits in the inner retina. Presynaptic inhibition onto retinal bipolar cell terminals regulates synapse organization at these terminals. (*Top*) Depiction of an adult rod bipolar cell (RBC) axon terminal providing glutamatergic input to A17 and AII amacrine cells (ACs) at ribbon synapses. The A17 provides GABAergic reciprocal feedback inhibition onto RBC terminals, which is mediated by GABA_A_α1 receptor (GABA_A_α1R)- and GABA_C_ receptor (GABA_C_R)-containing synapses. The RBC terminal also receives nonreciprocal GABAergic input. In the glutamic acid decarboxylase 67 (GAD67) knockout (KO), GABA synthesis is impaired and leads to a reduction of GABA_A_α1 receptors across RBC terminals (Schubert et al. 2013). When all vesicular release of inhibitory neurotransmitters in the retina is suppressed [vesicular inhibitory amino acid transporter (VIAAT) KO] (Hoon et al. 2015), both GABA_A_α1R and GABA_C_R clustering is impaired. Thus, the amount of presynaptic inhibition impinging on the RBC terminal regulates postsynaptic receptor clustering. (*Bottom*) The level of presynaptic inhibition not only regulates neurotransmitter release from the terminal, but also impacts selection of postsynaptic AC partners during assembly of the ribbon synapse dyads. In the VIAAT KO, when both GABA_A_ and GABA_C_ receptors are reduced at RBC terminals, errors in ribbon synapse assembly are noted with erroneous three-partner triad configurations and A17–A17 dyad profiles (Sinha et al. 2020). In the GABA_A_α3 KO, the formation of GABA_A_ synapses at RBC terminals is selectively impaired, and this also leads to deficits in RBC dyad assembly (erroneous monad and triadic profiles), although the extent of aberrations is less severe than in the VIAAT mutant (Sinha et al. 2021).

Glutamatergic transmission from BC terminals, however, is not necessary for the formation of inhibitory GABAergic synapses onto BC terminals (Schubert et al. 2013). Inhibitory neurotransmission onto BC terminals though does regulate the formation and maintenance of synapses mediating presynaptic inhibition in the inner retina (Schubert et al. 2013) (Figure 3). Interestingly, this regulation is synapse or receptor type specific and works in a dose-dependent manner to regulate the maintenance of inhibitory synapses across BC terminals (Hoon et al. 2015, Schubert et al. 2013). In retinas with impaired GABA synthesis, glutamate release from RBC terminals is transiently increased prior to eye opening, but the system compensates thereafter to ensure that RBC output matches expected levels (Schubert et al. 2013). These observations underscore a cross-dependency in the pathways regulating ribbon synapses and inhibitory synapses at BC terminals. Indeed, ultrastructural misorganizations in BC ribbon synapse assembly and postsynaptic partner selection are observed in mutants with impaired BC presynaptic inhibition (Sinha et al. 2020, 2021) (Figure 3), highlighting that the inhibition received by BC terminals regulates dyad circuit assembly.

The establishment of inhibitory synapses at the soma and dendrites of RGCs is also regulated by distinct activity-dependent mechanisms. In the DS circuit, reduction of GABAergic transmission increased the number and size of DS-RGC somatic GABA_A_R clusters, underscoring the ability of this synapse to compensate homeostatically for reduced presynaptic NT release (Bleckert et al. 2018). Interestingly, the dendritic GABA_A_R synapses on DS-RGCs remained unperturbed in this condition (Bleckert et al. 2018). This activity-dependent synaptic regulation during assembly of DS-RGC circuits is, however, interneuron specific, as suppressing GABA release from SACs alone does not impact somatic or dendritic DS-RGC receptor distributions (Bleckert et al. 2018).

Along the same lines, suppressing inhibitory neurotransmission from all retinal interneurons causes BC dendrites to increase GABA_A_R clustering, but suppression of neurotransmission from HCs alone does not alter receptor distribution at BC dendrites (Hoon et al. 2015). Inhibitory neurotransmission could thus operate in a dose-dependent and source-specific manner to regulate the development and organization of retinal inhibitory synapses.

Crosstalk between the axonal and dendritic BC compartments can regulate synaptic connectivity in some scenarios, whereas in others, these two BC compartments seem to be independently regulated. When the RBC population is reduced by >50%, RBC axons expand, and RBC output synapse density decreases, mirroring the alterations at the dendritic compartment and demonstrating a coregulation of synaptic connectivity between the input (dendritic) and output (axonal) BC compartments (Johnson et al. 2017). Lack of inhibitory neurotransmission, however, imposes distinct GABA_A_R synaptic alterations at the BC axonal versus dendritic compartment: Whereas the GABA_A_Rs at the dendritic compartment are upregulated following the expected trajectory for homeostatic compensation of postsynaptic receptors after presynaptic NT blockade (Hoon et al. 2015), the GABA_A_Rs at the BC axonal compartment downregulate receptor clustering, underscoring that compartment-specific regulatory mechanisms can tune retinal synaptic composition and function.

Another activity-dependent mechanism regulating synaptic connectivity in the inner retina is exposure to sensory stimuli. Dark-rearing alters global inner retinal interneuron activity, as determined by ERG recordings (Vistamehr & Tian 2004). Dark-rearing also prevents the maturation of RBC terminals, the accruing of receptor types and function at GABA synapses across these terminals, and the organization of the RBC ribbon output synapses (Wisner et al. 2023). At the RGC level, dark-rearing alters dendritic structure, spike responses, and synaptic properties (Tian & Copenhagen 2001, 2003). Dark-rearing additionally alters the molecular maturation of RGC types but does not impact the specification of RGC types (Whitney et al. 2023). Visual experience is required for the accurate dendritic orientation of a subset of DS-RGCs (El-Quessny et al. 2020), but the development of the DS circuit remains largely unaltered by dark-rearing, as direction-selectivity maps are conserved in dark-reared retinas (Tiriac et al. 2022). DS responses from RGCs, however, are abolished when the presynaptic AC type impinging on DS-RGCs (the SACs) is ablated (Yoshida et al. 2001). The impact of these activity manipulations on synaptic structures and connectivity remains unknown.

## 4. CONCLUDING REMARKS

The mechanisms instructing retinal synapse formation and function are diverse and pathway specific. Recent progress in the field of molecular organizers has widened our understanding of the role of the LRR family of proteins in regulating retinal synapses and demonstrated that distinct family members can be engaged at outer versus inner retinal synapses. In the outer retina, although a lot of work has been done on determining the molecular complexity that is responsible for photoreceptor→ON BC synapses, comparatively little is known about the molecular interactome operational for the organization and function of OFF BC synapses. In this regard, it is important to note that OFF CBC dendrites pool information from both rod and cone photoreceptors, although specific subtypes of OFF CBCs engage in the establishment of basal synapses at rod terminals (Behrens et al. 2016). Thus, distinct molecular patterns could not only be responsible for establishing ON versus OFF CBC synapses at cone terminals, but also be key for allocating OFF CBC dendrites to rod terminals. In the inner retina, given the diversity of AC and RGC types, an expansion of the complement of molecular organizers directing circuit assembly is likely.

Three-dimensional structural reconstructions of the molecular interaction domains would add considerably to our knowledge of the proteomic interactome directing retinal connectivity. As more isoforms of each protein family are being uncovered, it remains to be seen whether these have redundant or distinct (nonoverlapping) roles. This will determine the extent of functional redundancy that is built into retinal circuits. Given the diversity across retinal cell types, it will not be surprising if the field adds considerably to the list of molecular organizers regulating synapses in a (sub)type-specific manner. As we expand studies in the primate retina, we will be able to infer whether the roles of molecular organizers that we have observed in the murine retina are conserved across species. Notably, regional heterogeneities regulate circuit construction and visual function in the primate retina (Grünert & Martin 2020). Thus, a location-specific role of organizers and regulatory mechanisms for the primate retina is likely. Given the current expansion of transcriptomic databases available for retinal cell types, we should soon have information about the complete complement of organizers that establish retinal synapses. The ways in which activity imbalances impact the transcriptional signaling pathways are the basis for compensatory events in the retina, and given that these activity imbalances commonly occur during disease or degenerating conditions, the ability of the circuit to compensate is a critical area of research that needs to be expanded in future studies.

## SUMMARY POINTS

1. Activity-dependent mechanisms and molecular interactions drive synapse formation in the retina.
2. The leucine-rich repeat (LRR) family of proteins are key organizers for mammalian retinal circuits.
3. Organizers can be temporally regulated and can have retina-specific roles for synaptic formation.
4. The role of organizers can be cell type specific, pathway specific, and synapse type specific.
5. Organizers can engage in multiple interactions to mediate differential effects.
6. Activity imbalances impose disparate effects across retinal synapses.
7. Synaptic remodeling within the retinal circuit can preserve connectivity and functionality.
8. The retinal circuit can exhibit cellular and synaptic plasticity after retinal neuron loss and/or after removal of molecular organizers.

## FUTURE ISSUES

1. Intrinsic cues within specific retinal subtypes that direct connectivity and function are yet to be detailed.
2. Cues assembling OFF synapses in the outer retina remain to be detailed.
3. Correlated transcriptomic, functional, and ultrastructural analyses after activity perturbations or after loss of molecular organizers would provide increased knowledge of the synergistic mechanisms regulating retinal synapses.
4. Identification of the complete set of synaptic organizers for the diverse inner retinal synapse types is needed.
5. Proteomic interactions underlying retinal synaptic organization remain to be detailed.
6. Interspecies comparison is needed for delineation of conserved organizers and mechanisms across retinal circuits.
7. Mechanisms regulating circuit establishment within specialized regions of the mammalian retina, such as across the primate retina, are needed to understand the regional heterogeneities of organizers across retinal pathways.
8. Mechanisms of synaptic alterations and compensation after circuit perturbation and/or after cell degeneration are needed for a comprehensive understanding of the impacted pathways.
